# Assessment of gadoxetate DCE-MRI as a biomarker of hepatobiliary transporter inhibition

**DOI:** 10.1002/nbm.2946

**Published:** 2013-04-07

**Authors:** Jose L Ulloa, Simone Stahl, James Yates, Neil Woodhouse, J Gerry Kenna, Huw B Jones, John C Waterton, Paul D Hockings

**Affiliations:** a Science and Validation, Personalised Healthcare and BiomarkersAstraZeneca, Macclesfield, UK; b Molecular Toxicology, Safety Assessment UKAstraZeneca, Macclesfield, UK; c DMPK, Oncology iMedAstraZeneca, Macclesfield, UK; d Pathology, Safety Assessment UKAstraZeneca, Macclesfield, UK; e Science and Validation, Personalised Healthcare and BiomarkersAstraZeneca, Mölndal, Sweden; f MedTech West, Chalmers University of TechnologyGothenburg, Sweden

**Keywords:** drug-induced liver injury, DILI, DCE-MRI, liver, imaging biomarker, safety biomarker, hepatobiliary transporters, pharmacokinetic model

## Abstract

Drug-induced liver injury (DILI) is a clinically important adverse drug reaction, which prevents the development of many otherwise safe and effective new drugs. Currently, there is a lack of sensitive and specific biomarkers that can be used to predict, assess and manage this toxicity. The aim of this work was to evaluate gadoxetate-enhanced MRI as a potential novel biomarker of hepatobiliary transporter inhibition in the rat. Initially, the volume fraction of extracellular space in the liver was determined using gadopentetate to enable an estimation of the gadoxetate concentration in hepatocytes. Using this information, a compartmental model was developed to characterise the pharmacokinetics of hepatic uptake and biliary excretion of gadoxetate. Subsequently, we explored the impact of an investigational hepatobiliary transporter inhibitor on the parameters of the model *in vivo* in rats. The investigational hepatobiliary transporter inhibitor reduced both the rate of uptake of gadoxetate into the hepatocyte, *k*_1_, and the Michaelis–Menten constant, *V*_max_, characterising its excretion into bile, whereas *K*_M_ values for biliary efflux were increased. These effects were dose dependent and correlated with effects on plasma chemistry markers of liver dysfunction, in particular bilirubin and bile acids. These results indicate that gadoxetate-enhanced MRI provides a novel functional biomarker of inhibition of transporter-mediated hepatic uptake and clearance in the rat. Since gadoxetate is used clinically, the technology has the potential to provide a translatable biomarker of drug-induced perturbation of hepatic transporters that may also be useful in humans to explore deleterious functional alterations caused by transporter inhibition. Copyright © 2013 John Wiley & Sons, Ltd.

## INTRODUCTION

Drug-induced liver injury (DILI) is responsible for up to 15% of cases of acute liver failure in Europe and the USA and is one of the leading causes of drug attrition and/or delayed progression of new drug candidates 1,2. Underlying mechanisms are still poorly understood and are difficult to identify 2,3. For many drugs that cause DILI in man, liver injury is not evident in preclinical safety studies undertaken in animals, or in early clinical trials, but is first detected only during large and expensive Phase III trials or after the drug has reached the market and a very large number of patients has been exposed 4. Typically, the first indications of liver dysfunction are asymptomatic raised plasma levels of hepatic enzymes such as aspartate aminotransferase (AST) and alanine aminotransferase (ALT) 5. Although these signals are considered useful for identification of drugs that have the potential to cause DILI in the human population, it is important to recognise that they provide no information on the mechanisms that underlie DILI. In addition, for the majority of patients who exhibit hepatic enzyme elevations the liver dysfunction is transient and is not followed by symptomatic DILI, even if drug treatment is continued 4,5. However, a small proportion of patients who exhibit hepatic enzyme elevations may subsequently develop DILI, which in some instances can result in life threatening liver failure 3. Currently, there remains an unmet need for novel biomarker approaches that can provide useful mechanistic insights and, for patients who exhibit hepatic enzyme elevations, can distinguish between individuals who are at risk of DILI and those for whom the liver dysfunction is transient or compensatory.

In man, the most frequent observed patterns of DILI are hepatocellular (i.e. affecting hepatocytes), cholestatic (affecting bile flow and the biliary system) and mixed hepatocellular/cholestatic (where features of both hepatocellular and cholestatic injury are evident) 4,5. Hepatocellular DILI poses particular clinical concern, since for some drugs this may result in substantial loss of hepatocyte mass and development of liver failure. In contrast, the major clinical consequences of cholestatic DILI are chronic liver dysfunction arising from sustained impairment of bile flow, which may result in hepatic or biliary fibrosis, cirrhosis or vanishing bile duct syndrome 6,7.

**Figure 1:**
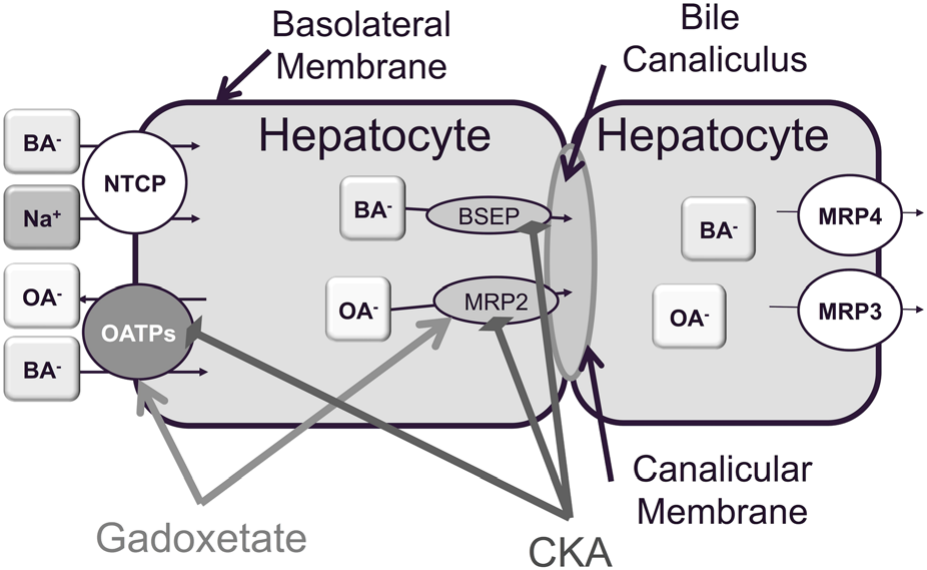
Overview of some of the uptake and efflux transporters expressed at the basolateral and canalicular membrane of hepatocytes. CKA, chemokine receptor antagonist compound; see text for details. OATP, organic anion transporting polypeptide; BA, bile acids; OA, organic anions; BSEP, bile salt export pump; MRP, multidrug resistance-associated protein; NTCP, Na^+^ taurocholate co-transporting polypeptide.

In recent years, an emerging body of evidence has highlighted the critical role played by hepatic transporters in uptake and biliary clearance of drugs and endogenous molecules (Fig. 1). Uptake of many drugs from blood plasma through the basolateral membrane into the hepatocyte is mediated by a family of multispecific organic anion transporting polypeptides (OATPs), which also mediate uptake of some bile salts and various metabolites 8,9. Many drugs have been found to inhibit the activities of hepatic OATP uptake transporters, and this process has the potential to cause drug–drug interactions that result in altered drug pharmacokinetics 10,11. In addition, drugs, their metabolites and numerous endogenous metabolites are secreted from hepatocytes into bile via specific ATP-dependent active transport processes such as the multidrug resistance-associated protein 2 (MRP2) 12,13. Genetically determined defects in MRP2 activity have been shown to result in elevated levels of conjugated bilirubin in plasma but not overt liver injury in humans (Dubin–Johnson syndrome) 14,15, and in some strains of rats 16,17. Bile salts are excreted into bile primarily by the bile salt export pump (BSEP), and genetically inherited defects in BSEP expression and BSEP activity result in cholestatic liver injury in humans 14,15. It has been observed that many drugs that cause cholestatic or mixed hepatocellular/cholestatic DILI inhibit BSEP activity 18–20. In view of this, it has been proposed that, in patients who develop liver injury caused by such drugs, inhibition of BSEP activity results in accumulation of bile salts within hepatocytes to cytotoxic levels, thereby leading to cholestatic liver injury 21.

In view of the observed association between hepatic biliary transport inhibition by drugs and increased risk of both drug–drug interactions and DILI, approaches that can be used to quantify transporter inhibition *in vitro* have been developed and are now starting to be utilised by drug developers. However, it is important to note that, at present, the effectiveness of this approach is limited by an incomplete scientific understanding of the relationship between biliary transporter inhibition *in vitro*, the functional consequences *in vivo* and the risk of developing DILI 19. This is due in large part to the restricted range of tools that are currently available to quantify hepatic transporter interactions *in vivo*, both in preclinical species and in man. The approaches used currently require highly invasive procedures (e.g. bile duct cannulation) or provide only indirect and mechanistically non-specific evidence of liver dysfunction (e.g. evaluation of plasma levels of bile salts, conjugated bilirubin, AST, ALT etc.). There is therefore an urgent need for improved *in vivo* approaches that can be used to study hepatobiliary transporter function. Non-invasive imaging techniques, such as MRI, are well placed to fill this gap.

Dynamic contrast-enhanced MRI (DCE-MRI) allows characterisation of functional aspects of physiology by adding a temporal dimension to the spatial information provided by MRI. DCE-MRI studies are normally performed using gadolinium-based contrast agents, which can be classified as extracellular (e.g. gadopentetate) or intracellular (e.g. gadoxetate), depending on their biodistribution in the body 22. Gadopentetate is a clinically approved ionic gadolinium chelate that after injection quickly distributes into the extracellular space and is completely cleared through the kidney 23,24. Gadoxetate is a clinically approved 25–27 hepatobiliary-specific MRI contrast agent 28–30. It is a lipophilic gadolinium chelate, which is excreted by both liver and kidney, in proportions that are species dependent 30. In rats, gadoxetate clearance is 70% biliary and 30% renal 28, while in humans 50% is excreted via each route 25. Gadoxetate utilises organic anion transport systems *in vivo*, since hepatic uptake is inhibited by bromosulfophthalein, bilirubin and rifampicin 29–32. *In vitro* experiments have shown that gadoxetate uptake is mediated by the liver-specific cell transporters human OATP1B1, OATP1B3 and Na^+^ taurocholate co-transporting polypeptide (NTCP) 20, and rat Oatp1a1 (previously called Oatp1) 33. *In vivo* experiments in TR^-^ rats have shown that biliary efflux of gadoxetate is mediated by Mrp2 31,34.

Gadoxetate has been used to measure liver function in rodent experimental models of liver fibrosis 35,36, fatty liver and bile duct obstruction 37,38. In rats, we have recently demonstrated that administration of estradiol 17-β-d-glucuronide, which is a well characterised cholestatic agent that impairs OATP, Mrp2 and Bsep function 9,20,39,40, transiently prolonged gadoxetate-induced MRI liver enhancement 39. In man, gadoxetate has been used successfully to detect and characterise liver lesions 41–46 because it is specifically taken up by hepatocytes but not tumour cells.

In the present study we have developed a novel model that describes the uptake of gadoxetate from the extracellular space into the hepatocyte and excretion into bile. We first used gadopentetate DCE-MRI to calculate the volume fraction of extracellular space in rat liver and spleen. This information was required by the model to enable estimation of the intracellular concentration of gadoxetate in the liver. We then used the model to quantify the hepatic uptake and excretion of gadoxetate in the rat, in the presence and absence of an investigational chemokine receptor antagonist (CKA) compound that we have found to inhibit biliary transporter activity *in vitro*. Our results indicate that this technique provides a novel approach for investigation of drug-induced perturbation of hepatic transporters *in vivo*.

## MATERIALS AND METHODS

### Animal preparation and imaging procedure

All experiments were conducted in compliance with licences issued under the UK Animals (Scientific Procedures) Act 1986 after review by the local ethics committee.

Male Han Wistar rats (232–340 g, Harlan, Bicester, UK) were fasted overnight and anaesthetised using 3% isoflurane in air. Anaesthesia was maintained at 1.5–2.0% throughout the imaging procedure. The tail vein was cannulated with a 26G catheter and flushed with heparin solution to prevent clotting (100 IU/ml in saline). Throughout the imaging procedure, rectal temperature and respiration rate were monitored (SA Instruments, New York, USA) and maintained at 37 °C and 50-60 breaths/min, respectively.

MRI was performed at 4.7 T (Oxford Instrument, Oxford, UK) incorporating a high power gradient set (200 mT/m, 121 mm inner diameter); Avance III, Bruker Biospin, Ettlingen, Germany, a 72 mm quadrature birdcage volume transceiver and Paravision 5.1 (Bruker Biospin). The imaging protocol included IntraGate FLASH 47 coronal images for anatomical reference of the abdominal cavity, an inversion recovery FISP 48,49 sequence for R1 mapping of the abdomen and IntraGate FLASH for DCE-MRI (see Table 1). Five minutes after the DCE-MRI acquisition commenced, the contrast agent (see following paragraphs) was administered intravenously (i.v.) via the tail vein with an automatic pump (5 ml/min, 305 piston pump, Gilson, Luton, UK).

**Table 1:** Details of the experiment design (MRI protocols)

	Localiser	R1 map	DCE-MRI
			
Sequence	IntraGate FLASH	IR-True FISP	IntraGate FLASH
Slices	10 × 2 mm	10 × 2 mm	10 × 2 mm
TR (ms)	60.3	3.4	60.3
TE (ms)	1.4	1.5	1.4
Flip angle (°)	30	4	30
FOV (mm^2^)	60 × 60	60 × 60	60 × 60
Matrix size (pixels)	256 × 256	128 × 128	256 × 256
TI (ms)	–	241 + 430*n* (*n* = 0, …, 14)	–
Time resolution (min/vol)	–	–	1

We first estimated the volume fraction of extracellular space in the liver. Five healthy rats (282 ± 9 g) were imaged following the protocol described in the previous paragraph and dosed i.v. with 100 µmol/kg of gadopentetate (Magnevist, Bayer Schering Pharma, Berlin, Germany). DCE-MRI data were continuously acquired for 45 min. After the imaging session, animals were euthanised with an overdose of isoflurane.

Second, we characterised the hepatic clearance of gadoxetate when co-administered with an inhibitor of biliary transporter activity 50. Thirty rats (body weight 298 ± 26 g), with six rats per group, were dosed orally with either the chemokine antagonist (CKA) 1-(4-chloro-3-trifluoromethyl-benzyl)-5-hydroxy-1-H-indole-2-carboxylic acid 51 or vehicle. The CKA was synthesised by AstraZeneca and had a purity of more than 99%. The CKA was formulated as suspensions in the vehicle (water containing 0.5% (w/v) hydroxypropyl-methylcellulose/0.1% (w/v) polysorbate 80) and was dosed by oral gavage at 20, 200, 500 or 2000 mg/kg.

Thirty minutes after administration of CKA or vehicle, animals were anaesthetised and imaged following the protocol described in Table 1. After a further 30 min (i.e. 60 min after the CKA or vehicle dose) DCE-MRI data acquisition commenced, and 5 min later 25 µmol/kg of gadoxetate (Primovist, Bayer Schering Pharma) was administered i.v. DCE-MRI data were acquired continuously for a total of 60 min. The start of DCE-MRI acquisition was defined as *t* = 0 min, injection of gadoxetate was *t* = 5 min and the end of DCE-MRI acquisition was *t* = 60 min. At the end of the imaging session, animals were euthanised with an overdose of isoflurane and blood and liver tissue samples were taken for analysis.

### Plasma chemistry analysis

Blood samples were taken from the *vena cava* into lithium-heparin tubes and the plasma fraction was separated by centrifugation at 1200 *g* and 4 °C for 10 min. Plasma samples were analysed on a Roche P Modular analyser (Roche Diagnostics, Burgess Hill, West Surrey, UK) using a total bilirubin assay, an alanine aminotransferase (ALAT/GPT) assay (both from Roche Diagnostics) and a total bile acid assay (Alere, Stockport, UK) according to the manufacturers’ instructions.

### Pathology analysis

At necropsy, 3–4 mm thick slices of the left lateral, right medial and caudate lobes of the liver were taken, immersed in 10% neutral buffered formalin and fixed for 48 h prior to standard tissue processing into paraffin wax. Sections (4–5 µm thick) were stained with haematoxylin and eosin (H&E) and examined by light microscopy.

### *In vitro* transporter analysis

#### Determination of inhibition of human OATP1B1 (hOATP1B1) activity

Recombinant cells expressing human OATP1B1 (HEK293-hOATP1B1) 52, prepared at the Department of Molecular Biology, AstraZeneca R&D (Charnwood, UK), were cultured in standard tissue culture plasticware in Dulbecco's modified Eagle medium (DMEM), containing 4.5 mg/ml glucose and GlutaMAX™ (Invitrogen, Paisley, UK) supplemented with 10% (v/v) heat-inactivated foetal calf serum (PAA Laboratories, Yeovil, UK) and 1 mg/ml geneticin (Invitrogen). To determine inhibition of hOATP1B1-mediated uptake of [^3^H]-estradiol 17β-glucuronide ([^3^H]-EG; specific activity = 50 Ci/mmol; PerkinElmer Life and Analytical Sciences, Amersham, UK), 150 000 cells/well were plated into BD Biocoat™ poly-d-lysine 24-well plates (BD Biosciences, Oxford, UK) and cultured for 2–3 days until 90% confluent. Prior to the assay, cells were washed three times with warm (37 °C) transport buffer (Hank's Balanced Salt Solution, 10 mM HEPES, pH 7.4) and pre-incubated for 15–30 min at 37 °C with transport buffer containing 3 or 30 μM CKA or dimethyl sulfoxide (DMSO) vehicle. The pre-incubation solution was replaced with transport buffer containing 20 nM [^3^H]-EG and 3 or 30 μM CKA or DMSO vehicle. In control reactions, CKA was replaced with 30 μM cyclosporine A to determine a value for 0% activity of hOATP1B1-mediated [^3^H]-EG uptake. The final concentration of DMSO was 0.5% (v/v) in all incubations. Each test condition was assessed in triplicate determinations. After 2 min, uptake was terminated by washing cells three times with ice-cold transport buffer and then adding 0.1% (v/v) Triton-X in water for at least 30 min to lyse the cells. The amount of [^3^H]-EG taken up by the cells was determined via liquid scintillation counting using a TRI-CARB 3170 (PerkinElmer Life and Analytical Sciences, Waltham, MA, USA). Effects on hOATP1B1-mediated [^3^H]-EG uptake were expressed as percentages relative to DMSO vehicle control (100%) after subtraction of background uptake in the presence of 30 μM cyclosporine A (0%). If the uptake activity in presence of test compound was below the cyclosporine A control, the activity was set to 0%.

#### Determination of inhibition of rat Bsep (rBsep) and rat Mrp2 (rMrp2) activity

rBsep (Abcb11) or rMrp2 (Abcc2) was expressed in *Spodoptera frugiperda Sf*21 insect cells from which membrane vesicles were prepared as described previously 19, with the modification that at the end of the preparation vesicles were re-suspended in 50 mM sucrose/10 mM HEPES/Tris pH 7.4. Abcc2 cDNA was obtained from Sprague Dawley rat liver and inserted into the pFastBac1 vector, and recombinant baculoviruses were generated with the Bac-to-Bac baculovirus expression system (Invitrogen). 100 µg vesicles were incubated with CKA or DMSO and the relevant substrate in transport buffer for 5 min at 37 °C. For rBsep 100 µg vesicles were incubated with 0.5 μM [^3^H]-taurocholate (PerkinElmer Life and Analytical Sciences, Waltham, MA, USA) and 5 mM ATP, 20 mM MgCl_2_, 7.7 mM HEPES/Tris pH 7.4, 141 mM KNO_3_, 157 mM sucrose, 12.5 mM Mg(NO_3_)_2_. For rMrp2, 100 µg vesicles were incubated with 10 μM 5^6^ carboxy 2',7'-dichlorofluorescein (CDF; Sigma-Aldrich, Poole, UK) and 5 mM ATP, 20 mM MgCl_2_, 7.5 mM HEPES/Tris pH 7.4, 141 mM KNO_3_, 157 mM sucrose, 5 mM magnesium d-gluconate hydrate, and 0.5 mM calcium gluconate. The rMrp2 stop buffer contained 50 mM sucrose, 100 mM KCl, 5 mM HEPES/Tris pH 7.4 and 5 mM EDTA. CDF uptake into vesicles was measured by determining the relative fluorescence units (RFU) using an EnVision™ multilabel reader (PerkinElmer Life and Analytical Sciences, Waltham, MA, USA) with *λ*_ex/em_ = 485/535 nm. IC_50_ values were calculated from normalised data (i.e. DMSO control set to 100% transport activity and maximum inhibition observed set to 0% transport activity) with non-linear regression for a sigmoidal dose–response using the four-parameter logistic equation in GraphPad Prism version 5.00 (GraphPad Software, San Diego, CA, USA).

### Calculation of gadoxetate concentration

In all the experiments, regions of interest (ROIs) covering the liver and spleen were manually selected using ImageJ 53, from both IR-FISP and DCE-MRI acquisitions, to calculate the mean longitudinal relaxation rate before contrast injection, *R*_1_(0), and to extract the time course, respectively. It was assumed that the *R*_1_ of each organ of interest was homogeneous within the whole organ, so *R*_1_(0) represents the mean pre-contrast longitudinal relaxation over the ROI. The mean post-contrast longitudinal relaxation over the ROI, *R*_1_(*t*), was calculated from the IntraGate FLASH images using the signal equation of a saturation recovery spoiled gradient echo 54:


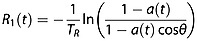
(1)

with *T*_R_ and *θ* the repetition time and flip angle of the IntraGate FLASH acquisition, respectively, and *a*(*t*) defined by 54


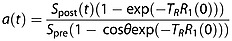
(2)

with *S*_pre_ and *S*_post_(*t*) the average signal intensity over the ROI of the baseline (i.e. pre-injection) and post-injection at a given dynamic time point, *t*, respectively.

It has been shown that in tumours 55,56 and organs such as skeletal muscle 57 and brain 58, the intercompartmental water exchange is not negligible, and the system departs from the fast exchange limit regime (FXL), in which case the relationship between *R*_1_(*t*) and the concentration of the contrast agent becomes non-linear 59–62. However, in the hepatocyte, the mean intracellular lifetime of a water molecule (*τ_i_* = 40–50 ms 63,64) and the water permeability coefficient (*P* = 66.4 × 10^-4^ cm/s 63) are comparable to the corresponding values in an erythrocyte (*τ_i_* = ∼10 ms, *P* = 50 × 10^-4^ cm/s), which is indeed in the FXL regime 57. Therefore the signal intensity can be converted to the concentration of extracellular contrast agent using the linear relationship 65


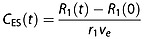
(3)

where *C*_ES_(*t*) represents the mean extracellular concentration of contrast agent across the ROI, *v*_e_ the volume fraction of extracellular space (ES) accessible by the contrast agent and *r*_1_ the relaxivity of the contrast agent. The relaxivities of gadoxetate and gadopentetate in liver and spleen at 4.7 T were assumed to be the same as in plasma and equal to 5.9 and 3.8 /s / mM, respectively 66.

Multicompartmental models have been used to describe the kinetics of extracellular Gd-based MRI contrast agents 55,59–62,67–69. Standard kinetic models usually consider three main compartments 69: plasma, extravascular extracellular space (EES) and intracellular compartments. However, in the case of a highly vascularised and perfused organ such as liver or spleen, where capillaries are highly permeable, the transfer exchange of contrast agent between plasma and EES occurs very fast (i.e. *K*^trans^ → ∞ 68), reducing it to two compartments: the extracellular space, comprising plasma and interstitial space 70,71, and the intracellular compartment, comprising hepatocytes and other liver cells, although we assume most of the intracellular space is occupied by hepatocytes 72. Following the reference region model approach 65, the extracellular concentration in the liver can be calculated by using another highly vascularised tissue, such as spleen, as the reference tissue.

For extracellular contrast agents the calculation of *C*_ES_ from the MRI signal in the organ of interest is straightforward using Equation [3] if *v*_e_ is known. *v*_e_ can be estimated as the area under the curve (AUC) ratio between plasma and tissue concentrations:


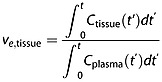
(4)

with *C*_plasma_(*t*) derived from the concentration of contrast agent in the whole blood, *C*_blood_(*t*), corrected for the haematocrit, Hct: *C*_plasma_(*t*) = *C*_blood_(*t*)/(1 − Hct). We have used this to measure the volume of extracellular space in liver and spleen with gadopentetate, which remains extracellular in these two tissues 71.

In later experiments examining gadoxetate uptake into hepatocytes we use the signal in the spleen to estimate *C*_ES_. Compared with measuring *C*_ES_ from the blood signal in a major artery or vessel, the spleen offers the advantage of higher signal to noise ratio, larger region of interest and reduced need to include saturation bands in the MRI acquisition. Therefore we use


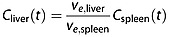
(5)

with *C*_liver_(*t*) and *C*_spleen_(*t*) the extracellular concentrations in liver and spleen, as derived from the MRI acquisition (i.e. *C*_tissue_(*t*) = *v*_e_*C*_ES_(*t*), where *C*_ES_(*t*) is the 'true' extracellular concentration in the tissue).

In the case of an intracellular tracer, the total concentration in the liver, *C*_t_(*t*), is the combination of the concentration in both the ES and intracellular (i.e. hepatocyte) compartments:


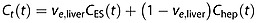
(6)

If the system is in the FXL regime, the total concentration is determined directly from the signal intensity by using Equation [3], and the intracellular component is determined from Equation [6]. However, in this work we propose a more general approach and calculate the intracellular concentration from the equivalent signal intensity as follows.

The total MRI signal (normalised to baseline) in a voxel, *S*(*t*), is made up of the contributions from each compartment 55:


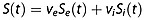
(7)

with *S*_e,i_(*t*) and *v*_e,i_ the signal intensity and measures of the water spaces in the extracellular (e) and intracellular (i) compartments, respectively (note that outside the FXL regime Equation [7] acquires a more complex form, with *v*_e_ and *v*_i_ (=1 − *v*_e_) replaced by *a*_S_ and *a*_L_ defined in 60).

The equivalent concentration in the hepatocyte, *C*_hep_(*t*), is obtained by solving Equations [1]–[3] for the intracellular component in Equation [7]. Given that the equivalent extracellular concentration in the liver, *C*_es_(*t*), is known (Equation [5]), the extracellular signal intensity in the liver is determined by solving backwards Equations [1]–[3]. The fraction of the signal intensity in the intracellular compartment can easily be determined if we know the total signal intensity in the liver, its fraction in the extracellular space and the fractional water spaces on each compartment.

### Non-linear compartmental model

Gadoxetate undergoes both renal (i.e. linear) and biliary (i.e. non-linear, Michaelis–Menten) elimination 28,73. In rats, the pharmacokinetics of gadoxetate plasma concentration can be well described by a model with central and peripheral compartments 28–30,32,74. This model assumes that elimination from a central plasma compartment follows first order kinetics in the kidney and Michaelis–Menten kinetics in the hepatobiliary system 28,29,74. In humans, a two compartment model was proposed with first order kinetics to describe gadoxetate uptake from the liver ES compartment into a hepatobiliary compartment consisting of hepatocytes and biliary ductules 75 with no efflux.

Here, we propose a two compartment model that follows the Tofts and Kermode formulation 67 to characterise the kinetics of gadoxetate clearance in the liver in terms of the rate of uni-directional uptake from the liver ES compartment into hepatocytes and the Michaelis–Menten constants of its efflux into bile.

**Figure 2:**
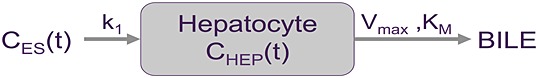
Two compartments that describe the kinetics of gadoxetate in the hepatocyte. *k*_1_ characterises the uptake from the extracellular space into the hepatocyte and the Michaelis–Menten constants *V*_max_ and *K*_M_ characterise the gadoxetate efflux from the hepatocyte into bile.

We can therefore isolate the intracellular compartment and characterise gadoxetate kinetics in the hepatocyte (Fig. 2) using the concentrations in each compartment derived in the previous section. The model defined in Figure 2 is expressed mathematically by a non-linear differential equation 73,74:


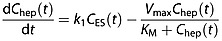
(8)

with *C*_hep_(0) = 0, *k*_1_ the uptake rate of gadoxetate from the extracellular space into the hepatocyte (i.e. Oatp1a1), and the Michaelis–Menten constants of biliary efflux *V*_max_ (the maximum rate of efflux into bile) and *K*_M_ (the concentration of gadoxetate when the rate of efflux is half the maximum rate, i.e. *V*_max_/2). These three parameters reflect the effect of the CKA on the kinetics of gadoxetate transport 76, and they are estimated for each dose group by solving the following minimisation problem:


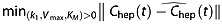
(9)

*C*_*hep*_(*t*) is determined by solving Equation [8] with the initial condition *C*_hep_(0) = 0, 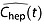 represents the actual measurement from the MRI data and || · || the second norm operator. Note the non-negativity constraint in all three parameters.

Parameter estimation was performed in MATLAB (R2011a, MathWorks, Natick, MA, USA) using the optimization and differential equation toolboxes.

### Statistical analysis

The significance of the effect of the CKA on the kinetics of gadoxetate uptake (*k*_1_) and clearance (*V*_max_ and *K*_M_) was investigated via ANOVA analysis 77 of the logarithm of each parameter, where variability was expressed in 95% confidence limits (CLs). Normality and homoscedasticity (i.e. variance homogeneity) of the data were confirmed via the Shapiro–Wilk normality test 78 and the Bartlett test of homogeneity of variances 79, respectively (95% CL). When comparing multiple sample groups in which marked changes were detected (95% CL), a multicomparison Tukey test 80 was performed to identify which of these differences were statistically significant. The directions of the changes were assessed with a one tailed *t*-test for the means between pairs of adjacent groups.

All the statistical analyses were performed in R v2.13.0 81, using the *stats* and *agricolae* (http://tarwi.lamolina.edu.pe/∼fmendiburu/) packages.

## RESULTS

### Estimation of the volume fraction of extracellular space in liver

**Figure 3:**
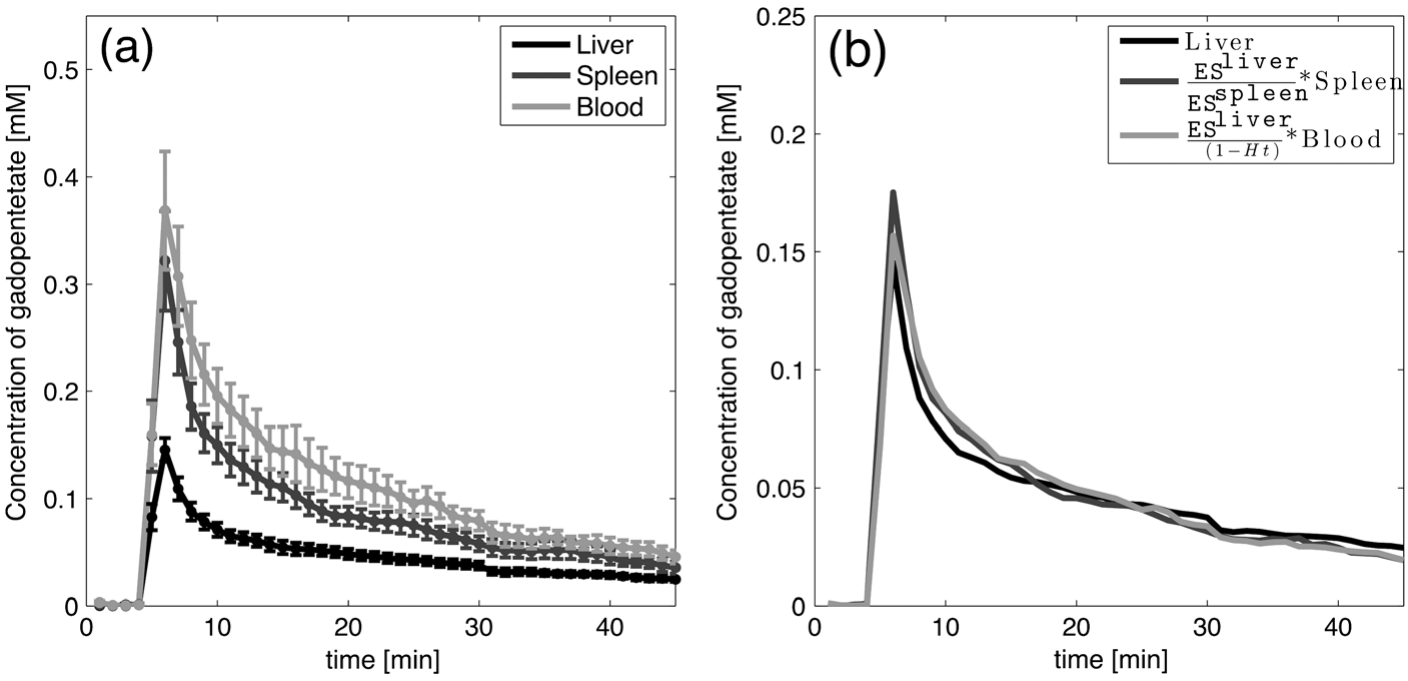
(a) Concentration of gadopentetate in liver, spleen and blood; solid lines represent the mean concentration and the bars the SEM over the five animals. (b) Concentration of gadopentetate in ES in liver, equivalent signal scaled from the spleen data and, for reference, the equivalent signal scaled from blood data.

Figure 3(a) shows the mean gadopentetate concentration data over time (five animals) in liver, spleen and blood following i.v. administration of the contrast agent. Using Equation [4] and a haematocrit value of 0.45, the estimated liver and spleen fractional extracellular spaces were equal to 0.23 ± 0.02 and 0.43 ± 0.02 (mean ± standard error of the mean (SEM)), respectively (*p* < 0.001 for the difference in extracellular spaces). Figure 3(b) shows that the liver gadopentetate time course can be represented by the spleen data scaled to an equivalent ES value. The blood data are included for reference.

### Inhibition of transporter activity *in vitro*

**Figure 4:**
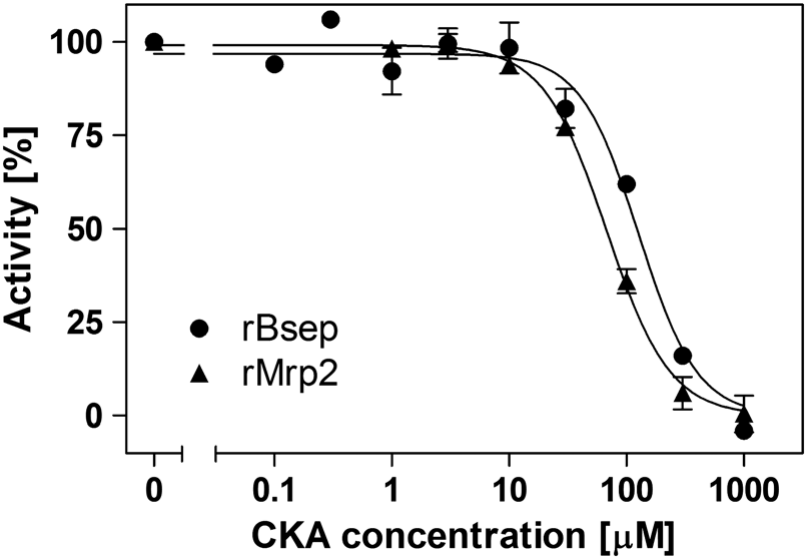
Inhibition of rBsep and rMrp2 transport activity by the CKA in membrane vesicles *in vitro*. Data are mean ± SEM of five (rBsep) or four (rMrp2) separate test occasions.

Inhibition of human OATP1B1 (hOATP1B1) was determined in a stably transfected HEK293 cell line that overexpressed the transporter using the probe substrate [^3^H]-estradiol 17β-glucuronide ([^3^H]-EG) 52. At concentrations of 3 and 30 μM CKA, the remaining hOATP1B1-mediated [^3^H]-EG uptake activity was 1% and 0% (mean of one experiment per test condition performed in triplicate), respectively, i.e. the activity of the transporter was completely inhibited. The effects of the CKA on ATP-dependent transporter activity of rat Bsep (rBsep) and rat Mrp2 (rMrp2) were determined using membrane vesicle assays and transporter selective probe substrates (Fig. 4). The CKA inhibited [^3^H]-taurocholate uptake into vesicles expressing rBsep with an IC_50_ value of 129.7 μM (95% CL of geometric mean 109.8–153.2; *n* = 5) and CDF uptake into vesicles expressing Mrp2 with an IC_50_ value of 68.5 μM (95% CL of geometric mean 58.2–80.7; *n* = 4).

### Evaluation of effects of CKA on hepatic clearance of gadoxetate *in vivo*

**Figure 5:**
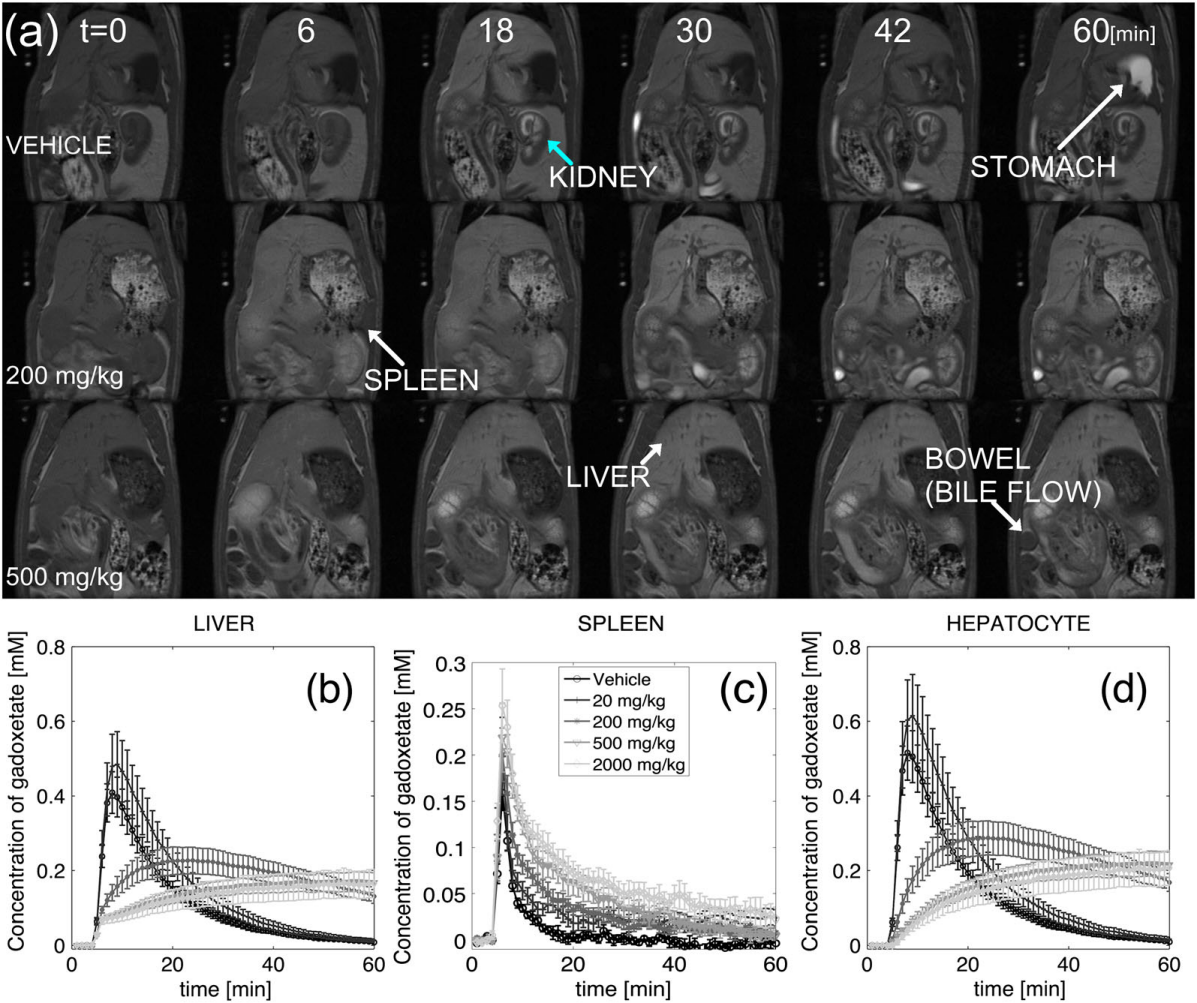
(a) Examples of dynamic images for animals treated with vehicle (top), 200 mg/kg (middle) or 500 mg/kg (bottom) CKA at *t* = 0, 6, 18, 30, 42 and 60 min after contrast injection. Note the enhancement of the small bowel lumen at about 30 min after contrast injection and also the reflux of gadoxetate into the stomach at the end of the acquisition in the vehicle treated animal. No enhancement was observed in the bowel of the animal treated with 500 mg/kg CKA. (b)–(d) Mean concentration of gadoxetate over ROIs covering (b) liver, (c) spleen and (d) hepatocytes. Bars represent SEM.

Figure 5(a) shows representative images from three rats treated by oral gavage with vehicle, CKA at 200 mg/kg or CKA at 500 mg/kg, at baseline (*t* = 0) and five time points after subsequent i.v. injection of the contrast agent gadoxetate (*t* = 6, 18, 30, 42 and 60 min). Compared with animals treated with vehicle, a marked and dose-dependent reduction in the rate of gadoxetate uptake into the liver and clearance from the liver into the GI tract was evident in animals treated with 200 and 500 mg/kg CKA. It is especially notable that enhancement of the small bowel lumen and also reflux of gadoxetate into the stomach was observed in the vehicle-treated animal. In contrast, no enhancement of the bowel was detected in the animal treated with 500 mg/kg CKA.

Figure 5(b)–(d) shows the time course of the gadoxetate concentration in the liver, spleen and hepatocytes as determined by Equation [9] (time courses are the mean over the ROI within each group and error bars represent SEM).

As can be seen in Figure 5(c), after about 30 min the amplitude of the spleen signal was comparable to the noise level in the vehicle- and lower-dose-treated animals. In order to minimise the contribution of this noise to the parameter estimation, a bi-exponential function 67 was fitted to the spleen data and used as *C*_spleen_(*t*) in Equation [5]. The concentration of gadoxetate in the hepatocyte (Figure 5(d)) was derived from its equivalent signal intensity using Equation [3]. The signal intensity in the hepatocyte was calculated from the signal intensity in liver and spleen (measured from the gadoxetate data) and the volume fraction of ES (calculated from the gadopentetate data) using Equation [7].

**Figure 6:**
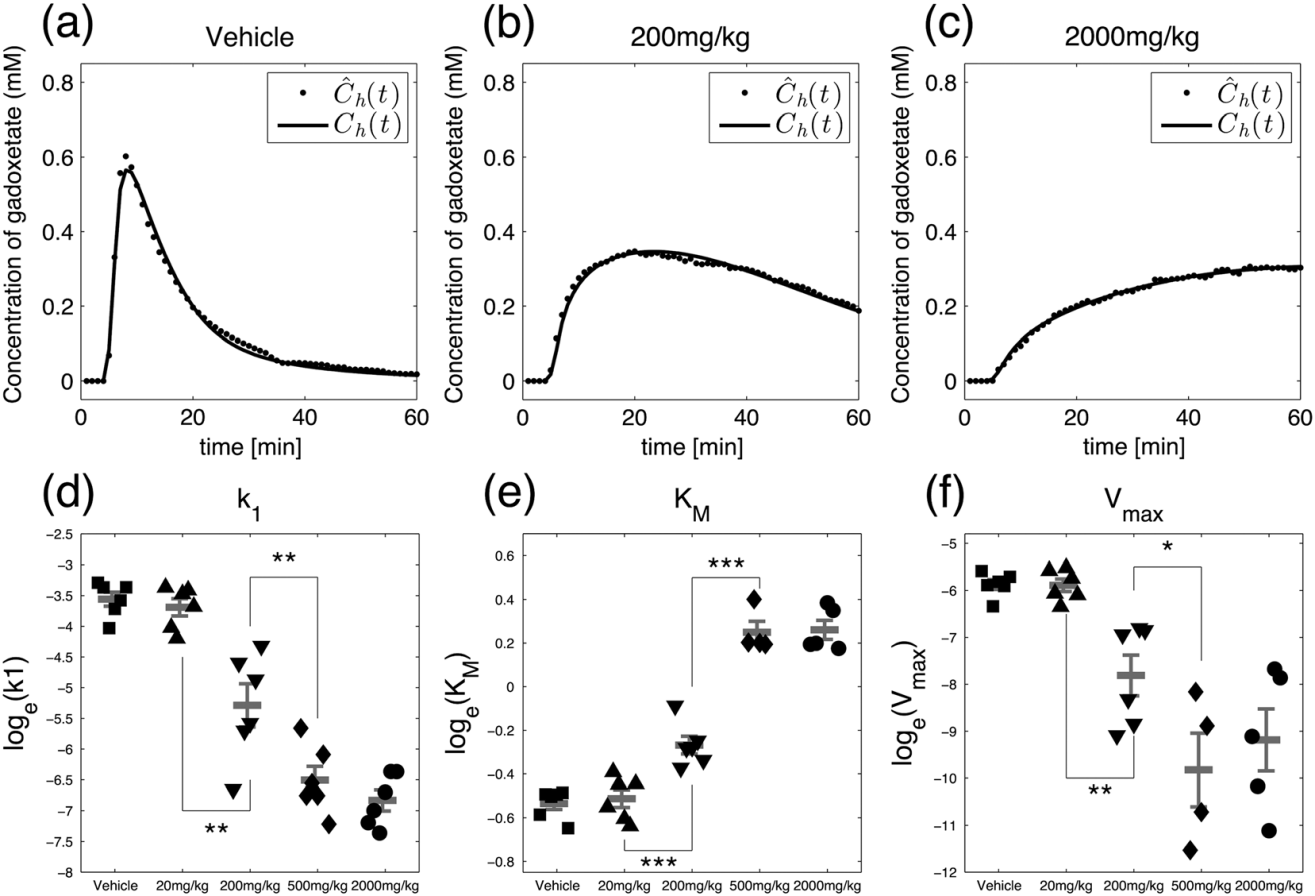
(a)–(c) Output of the pharmacokinetic model of hepatic clearance of gadoxetate (solid line) compared with the actual data extracted from the MR images (dotted line) for three representative animals treated with (a) vehicle, (b) 200 mg/kg CKA or (c) 500 mg/kg CKA. (d)–(f) Parameters of the model for individual animals in each group: (d) rate of uptake into the hepatocyte, *k*_1_, (e) rate of efflux into bile, *V*_max_, and (f) Michaelis–Menten constant, *K*_M_. Each symbol represents an individual animal; mean ± SEM is also given for each group. Significance codes: *** *p* < 0.001, ** *p* < 0.01, * *p* < 0.05.

In Figure 6(a)–(c), representative plots of the fitting results obtained from rats treated with vehicle, or with CKA at 200 mg/kg or 500 mg/kg, are shown. The calculated rate of uptake into hepatocytes, *k*_1_, and the Michaelis–Menten constants, *V*_max_ and *K*_M_, for its biliary excretion in all dose groups are summarised in Figure 6(d)–(f) and are listed in Table 2. The data analysis showed no significant effect on the kinetic parameters caused by CKA administration at 20 mg/kg (*p* = 0.23, 0.46 and 0.44 for the differences compared with vehicle in *k*_1_, *V*_max_ and *K*_M_, respectively), whereas all three parameters were altered in rats dosed with CKA at 200 mg/kg, 500 mg/kg or 2000 mg/kg. In particular, there was a marked dose-dependent reduction in *k*_1_ and *V*_max_ from 20 mg/kg to 200 mg/kg (*p* = 0.002 and 0.003 for *k*_1_ and *V*_max_, respectively) and from 200 mg/kg to 500 mg/kg (*p* = 0.009 and 0.037 for *k*_1_ and *V*_max_, respectively). No significant differences were observed between the changes in all three parameters in rats dosed with CKA at 500 mg/kg and 2000 mg/kg (*p* = 0.14, 0.56 and 0.33 for the differences in *k*_1_, *V*_max_ and *K*_M_, respectively).

**Table 2:** Summary of the pharmacokinetic parameters that characterise the hepatic clearance of gadoxetate (mean ± SEM). Gadoxetate accumulates in the liver because the uptake rate, *k*_1_, is one to two orders of magnitude larger than the efflux rate, *V*_max_, producing liver enhancement in the MR images

Groups	*k*_1_ (µmol/s)	*V*_max_ (µmol/s)	*K*_M_ (μM)
Vehicle	29.4 ± 3.0	2.9 ± 0.3	586.8 ± 15.4
20 mg/kg	26.1 ± 3.3	2.9 ± 0.4	601.3 ± 24.2
200 mg/kg	6.6 ± 1.9	0.6 ± 0.2	768.0 ± 32.4
500 mg/kg	1.7 ± 0.4	0.1 ± 0.1	1288.6 ± 68.1
2000 mg/kg	1.2 ± 0.2	0.1 ± 0.1	1302.8 ± 58.3

### *In vivo* clinical chemistry and pathology findings

**Figure 7:**
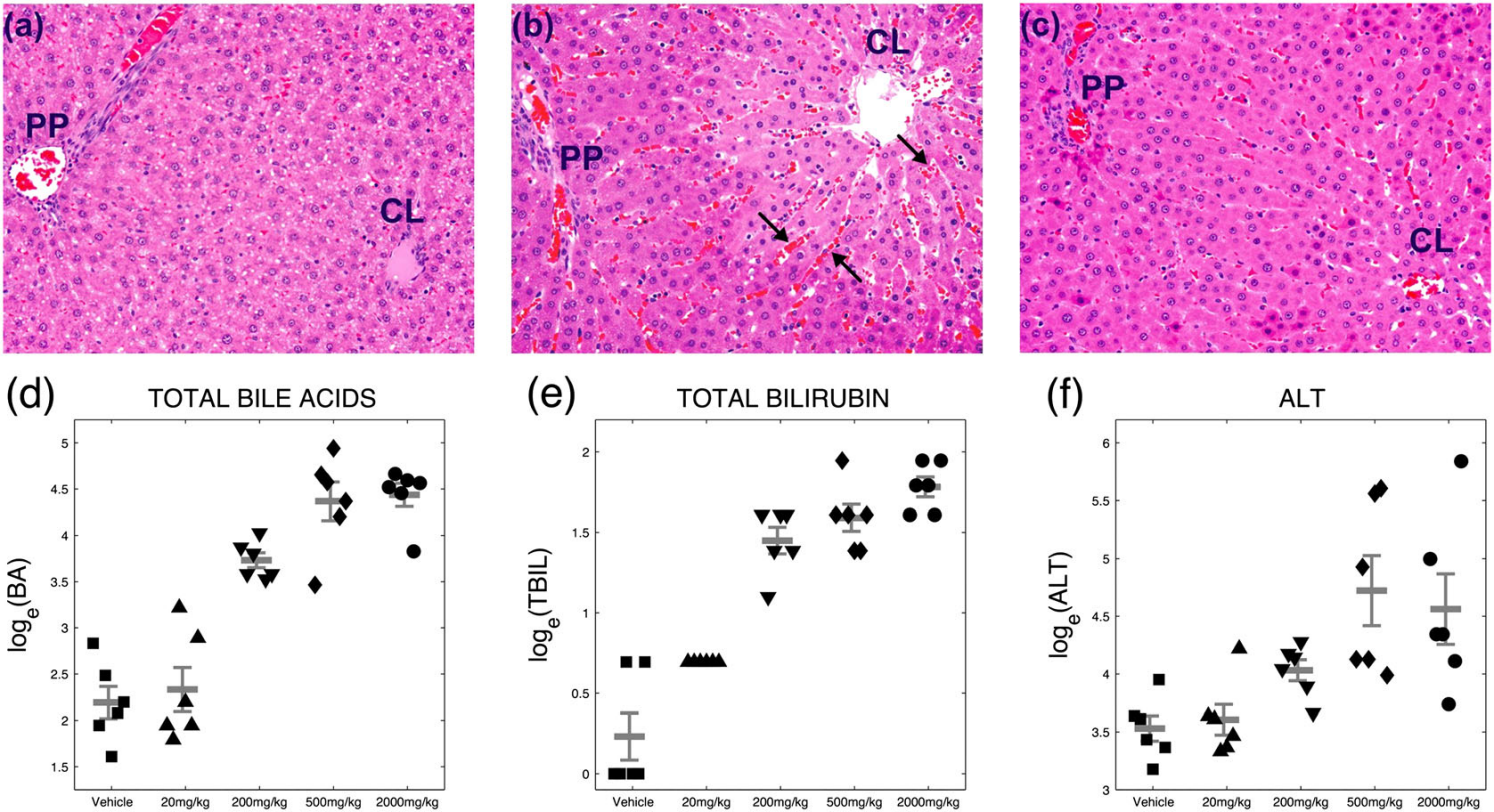
(a)–(c) Images of liver sections stained with haematoxylin and eosin: (a) vehicle-treated animal, (b) animal treated with 2000 mg/kg CKA showing centrilobular hepatocellular degeneration and necrosis (pale area) and sinusoidal congestion (arrows) and (c) animal treated with 2000 mg/kg CKA without drug induced findings. CL, centrilobular; PP, periportal. (d)–(f) Plasma chemistry parameters for individual animals in each group: (d) total bile acids, BA, (e) total bilirubin, TBIL, and (f) alanine aminotransferase, ALT. Mean ± SEM are also given for each group.

Evaluation of H&E-stained liver sections by light microscopy (Figure 7(a)–(c)) demonstrated that only one out of six animals dosed with 2000 mg/kg CKA exhibited significant centrilobular hepatocellular degeneration and necrosis, which was accompanied by neutrophil infiltration and associated sinusoidal congestion (Figure 7(b)). This was observed in the right median lobe section only and is illustrative of acute intoxication. Other liver lobe tissue sections from this animal resembled those from all other animals in this group (e.g. Figure 7(c)) and did not show any overt changes attributable to CKA treatment. Liver sections from rats given CKA at doses of 20, 200 or 500 mg/kg displayed no histological features indicative of drug-induced hepatotoxicity.

Increases in plasma markers indicative of functional hepatobiliary impairment, i.e. total bilirubin and bile acids, exhibited marked dose-dependent increases following administration of CKA (Figure 7(d),(e)). There was also a dose-related increase in plasma ALT levels in CKA-treated animals, indicating enzyme leakage from damaged liver cells, although data for individual animals were more variable than for other plasma chemistry parameters, and this was most evident in the groups treated with 500 and 2000 mg/kg CKA (Figure 7(f)).

**Figure 8:**
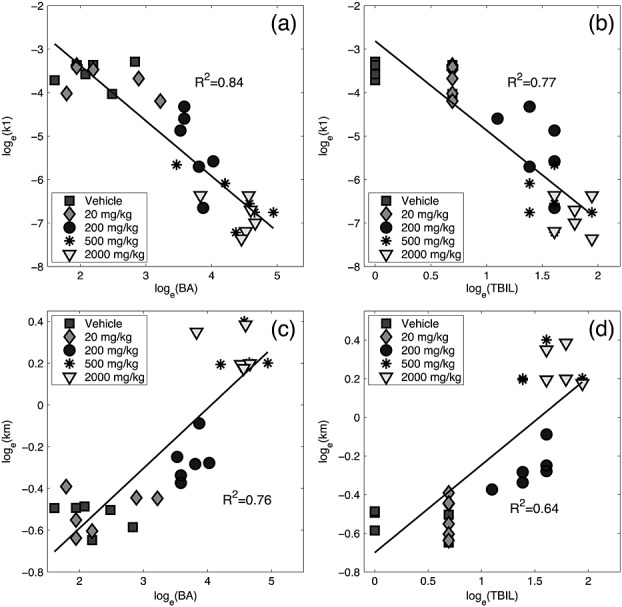
(a, b) Correlation of gadoxetate *k*_1_ and plasma marker concentrations of (a) total bile acids, BA, and (b) total bilirubin, TBIL. (c, d) Correlation of gadoxetate KM and plasma marker concentrations of (c) total bile acids, BA, and (d) total bilirubin, TBIL. Each symbol represents an individual animal.

A striking correlation was evident between CKA-induced elevations in plasma bile acids and total bilirubin and the effects of CKA administration on kinetic parameters of hepatic uptake and biliary excretion of gadoxetate (Fig. 8). In contrast, there was no correlation between the observed increases in plasma ALT levels and the hepatic uptake rate *k*_1_ (*r*^2^ = 0.44) or the biliary efflux *K*_M_ (*r*^2^ = 0.41).

## DISCUSSION

Our investigations have shown that DCE-MRI with gadoxetate provides highly robust and reproducible data to evaluate hepatic transporter function in anesthetised rats *in vi*vo. We have also presented a novel non-linear two compartment model that describes the kinetics of hepatic uptake and biliary excretion of gadoxetate in the hepatocyte.

Gadoxetate is formed by including the lipophilic ethoxybenzyl moiety (EOB) to the extracellular MRI contrast agent gadopentetate, which results in hepatocyte-specific uptake of gadoxetate 28,33. When constructing the model, we therefore assume that gadoxetate does not access the intracellular space of non-hepatocytes, and that its concentration in the extracellular space of the liver is equivalent to its concentration in the extracellular space of other organs (e.g. spleen) 33,34,75. The relative volume fraction of extracellular space between liver and spleen was estimated by using gadopentetate. We then used this information to estimate the intracellular, i.e. hepatocyte, concentration of gadoxetate and to determine the parameters of the non-linear model that enabled us to characterise its hepatic uptake and excretion kinetics.

The results we have obtained indicate that this model provides a simplified but effective description of hepatic transport of gadoxetate. The parameters provided by the model were its rate of hepatocyte uptake (*k*_1_) and the Michaelis–Menten values for biliary efflux (*K*_M_ and *V*_max_). Using this approach, we have been able to explore the use of gadoxetate DCE-MRI to investigate functional effects on hepatobiliary transporter function *in vivo*. We have used a CKA test compound, which was found to inhibit the hepatobiliary transporters OATP1B1 (the human orthologue of rat Oatp1a1), Mrp2 and Bsep *in vitro*.

Although the DCE-MRI imaging data cannot in themselves provide definitive insight into the mechanism of transporter inhibition, it is clear that both a marked dose-dependent reduction of gadoxetate uptake into the liver and efflux from the hepatocyte are observed in the images in the presence of the CKA. The dose-dependent inhibition of hepatic uptake by the CKA, represented by *k*_1_, is indicative of inhibition of organic anion uptake transporter proteins, most likely Oatp1a1, since gadoxetate was previously identified as a substrate for this transporter 21. Although it is unknown which of the rodent Oatps is the counterpart of human OATP1B1, members of the OATP/Oatp family have similar substrate and inhibitor specificities, with some exceptions 82,83. It is therefore conceivable that the inhibitory effect of the CKA on hOATP1B1, which we observed *in vitro*, also applies to the rat ortholo.gue *in vivo*. The MRI signal enhancement observed in the liver is a consequence of accumulation of gadoxetate in hepatocytes due to reduced efflux into bile caused by inhibition of Mrp2 by the CKA. These *in vivo* data are supported by the *in vitro* Bsep and Mrp2 inhibition data obtained and by a report that gadoxetate is a substrate of Mrp2 34. The calculated increase in the *K*_M_ of biliary efflux of gadoxetate is suggestive of competitive inhibition by the CKA 76.

The observed effects of the CKA on the kinetics of hepatic clearance of gadoxetate are consistent with previous studies in rats, where administration of OATP inhibitors such as bromosulfophthalein, rifamycin, bilirubin, prednisolone, doxorubicin hydrochloride, cisplatin, propranolol hydrochloride and rifampicin resulted in a reduction of maximum hepatic MRI signal enhancement 28,29,84–87. This highlights the potential of gadoxetate MRI to be a useful tool for evaluation of the drug–drug interaction potential of pharmaceuticals. In particular, drugs that interact with members of the OATP family have been demonstrated to have clinically relevant interactions leading to altered pharmacokinetics and potentially toxicity 11,88. Although a recent study in healthy volunteers did not show a statistically significant difference in relative liver MRI signal enhancement in subjects receiving the OATP inhibitor erythromycin compared with control subjects 89, the concept has not yet been explored with other clinically relevant drugs in man. This approach has potential benefit in the early phases of clinical trials when a liver safety signal is seen that is consistent with hepatobiliary transport inhibition and subsequent cholestasis, but it is not clear whether the signal is transient and compensation will occur or whether the patient or volunteer will progress to liver damage. In this situation an assay of *in vivo* hepatobiliary transporter function could enable more effective decision making.

Given the short duration of the study, overt cellular damage is not expected at this time point, which is reflected in a relatively poor correlation observed between the effects of CKA administration on gadoxetate hepatic transport kinetics and plasma ALT levels. Elevated serum levels of ALT reflect leakage of the enzyme from damaged cells (primarily hepatocytes) and not hepatobiliary transporter function. In fact, only a single animal in the study (dosed with CKA at 2000 mg/kg) showed necrosis and centrilobular hepatocellular degeneration (i.e. cellular damage) together with a markedly elevated plasma ALT value (344 IU/l). In contrast, we observed a close correlation between the effects of CKA compound administration on gadoxetate hepatic transport kinetics and plasma markers of hepatobiliary clearance, i.e. levels of bile acids and bilirubin. This finding provides further confirmation that administration of CKA inhibits hepatobiliary transporter function *in vivo* and is consistent with the data demonstrating inhibition of Mrp2 and Bsep by the compound *in vitro*. It also demonstrates that gadoxetate DCE-MRI is a much more sensitive technique for exploration of functional hepatobiliary transport inhibition than liver histopathology, as is evaluation of plasma levels of bile acids and bilirubin. It must be noted that, although the acquisition of DCE-MRI data was started one hour before the blood samples were taken, they strongly correlated with a decrease in the hepatic uptake rate *k*_1_. This might suggest that gadoxetate DCE-MRI could be an earlier biomarker of impaired hepatic transporter activity than soluble plasma markers.

The plasma bile acid elevations caused by CKA administration can be attributed to the known inhibitory effect of the CKA on Bsep activity and have been observed with other drugs 90–92. These data also raise the intriguing possibility that inhibition of Bsep activity *in vivo* by the CKA administration might have contributed to the observed inhibition of biliary gadoxetate excretion, but possible transport of gadoxetate by Bsep has not been investigated. The elevated plasma bilirubin levels are likely to be a consequence of inhibition of hepatic uptake of bilirubin and its glucuronide form via OATPs 93,94 and of inhibition of biliary excretion of conjugated bilirubin by Mrp2 95.

In recent years, evidence has been accumulated that suggests that impaired function of hepatobiliary transporters such as BSEP and MRP2, either through drug-induced inhibition or genetically determined reduction of activity, plays a role in drug-induced liver injury 19,96,97. Furthermore, inhibition of OATPs and MRP2 is associated with drug-induced hyperbilirubinaemia 98,99, and drug–drug interactions related to certain OATPs have clinical relevance 11. Consequently, candidate drugs are frequently assessed for transporter interactions during drug discovery using *in vitro* approaches. A major challenge when attempting to understand the functional significance of such *in vitro* data is that the relationship between inhibition of transporter activity by drugs *in vitro* and potential deleterious functional effects *in vivo* remains ill defined 100. This highlights the urgent need for new approaches that can be used to generate such understanding. Our results suggest that gadoxetate DCE-MRI may be a suitable technique for this purpose, especially where this is complemented by evaluation of bile acids and bilirubin as plasma biomarkers. In the future it will be important to generate data in animals treated with a broad range of different test compounds exhibiting a range of potency and selectivity of transport inhibition in order to develop a more complete understanding of the consequences that may ensue *in vivo*. In addition, gadoxetate MRI has been used extensively in the clinic 41–46, which raises the possibility that the approach might also be useful during drug development, to aid in clinical hazard identification and risk assessment of novel candidate drugs.

## CONCLUSION

This study has shown that gadoxetate DCE-MRI enables quantification of drug-induced alterations in hepatobiliary transporter activity in the rat *in vivo*. Administration to rats of a test compound (CKA) that inhibited the activity of the hepatic transporters OATP, Bsep and Mrp2 *in vitro* resulted in changes in the kinetics of gadoxetate clearance that could be explained in terms of effects on three parameters: the rate of gadoxetate uptake into hepatocytes, *k*_1_, and the Michaelis–Menten constants for efflux from hepatocytes into bile, *V*_max_ and *K*_M_. These changes correlated closely with effects of CKA compound on serum biomarkers indicative of biliary transporter inhibition (total bile acids and bilirubin). We conclude that gadoxetate DCE-MRI can detect inhibition of hepatobiliary transporters and has the potential to serve as a novel biomarker of functional cholestasis. Furthermore, since gadoxetate is used in the clinic, it provides a novel approach that is potentially applicable both in laboratory animals and in patients in order to support safety evaluation of novel pharmaceuticals.

## Abbreviations

ALT: alanine aminotransferase
AST: aspartate aminotransferase
Bsep: bile salt export pump
CKA: chemokine receptor antagonist
DCE-MRI: dynamic contrast-enhanced MRI
DILI: drug-induced liver injury
DMSO: dimethyl sulfoxide
EES: extravascular extracellular space
ES: extracellular space
Mrp2: multidrug resistance-associated protein 2
OATP: organic anion transporting polypeptide
SEM: standard error of the mean.
